# An Innovative Faster R-CNN-Based Framework for Breast Cancer Detection in MRI

**DOI:** 10.3390/jimaging9090169

**Published:** 2023-08-23

**Authors:** João Nuno Centeno Raimundo, João Pedro Pereira Fontes, Luís Gonzaga Mendes Magalhães, Miguel Angel Guevara Lopez

**Affiliations:** 1 Instituto Politécnico de Setúbal, Escola Superior de Tecnologia de Setúbal, 2914-508 Setúbal, Portugal; joao.raimundo20@estudantes.ips.pt; 2 Centro ALGORITMI, Universidade do Minho, Campus de Azurém, 4800-058 Guimarães, Portugal; id8968@uminho.pt (J.P.P.F.); lmagalhaes@dsi.uminho.pt (L.G.M.M.)

**Keywords:** breast cancer detection, magnetic resonance imaging, computer vision, machine learning, deep learning, convolutional neural networks

## Abstract

Replacing lung cancer as the most commonly diagnosed cancer globally, breast cancer (BC) today accounts for 1 in 8 cancer diagnoses and a total of 2.3 million new cases in both sexes combined. An estimated 685,000 women died from BC in 2020, corresponding to 16% or 1 in every 6 cancer deaths in women. BC represents a quarter of a total of cancer cases in females and by far the most commonly diagnosed cancer in women in 2020. However, when detected in the early stages of the disease, treatment methods have proven to be very effective in increasing life expectancy and, in many cases, patients fully recover. Several medical imaging modalities, such as X-rays Mammography (MG), Ultrasound (US), Computer Tomography (CT), Magnetic Resonance Imaging (MRI), and Digital Tomosynthesis (DT) have been explored to support radiologists/physicians in clinical decision-making workflows for the detection and diagnosis of BC. In this work, we propose a novel Faster R-CNN-based framework to automate the detection of BC pathological Lesions in MRI. As a main contribution, we have developed and experimentally (statistically) validated an innovative method improving the “breast MRI preprocessing phase” to select the patient’s slices (images) and associated bounding boxes representing pathological lesions. In this way, it is possible to create a more robust training (benchmarking) dataset to feed Deep Learning (DL) models, reducing the computation time and the dimension of the dataset, and more importantly, to identify with high accuracy the specific regions (bounding boxes) for each of the patient’s images, in which a possible pathological lesion (tumor) has been identified. As a result, in an experimental setting using a fully annotated dataset (released to the public domain) comprising a total of 922 MRI-based BC patient cases, we have achieved, as the most accurate trained model, an accuracy rate of 97.83%, and subsequently, applying a ten-fold cross-validation method, a mean accuracy on the trained models of 94.46% and an associated standard deviation of 2.43%.

## 1. Introduction

Replacing lung cancer as the most commonly diagnosed cancer globally, Breast Cancer (BC) today accounts for 1 in 8 cancer diagnoses and a total of 2.3 million new cases in both sexes combined [1]. An estimated 685,000 women died from BC in 2020, corresponding to 16% or 1 in every 6 cancer deaths in women. BC represents a quarter of a total of cancer cases in females and by far the most commonly diagnosed cancer in women in 2020 [2]. However, early detection and accurate diagnosis are significant to improve the prognosis and increase the survival rate of patients with BC by 30% to 50% [3]. The treatment of BC is highly effective when it is detected in the early stages of the disease [4]. Therefore, the early detection of BC is a critical issue that represents an urgent global priority. In the coming years, countries with weak health systems and lower incomes will suffer more severe consequences in terms of both diagnosis and mortality related to BC [5]. The COVID-19 pandemic severely hampered the process of cancer diagnosis and treatment at a worldwide level, e.g., developed countries such as Canada, the Netherlands, Germany, Italy, the United Kingdom, and Australia even suspended their national BC screening programs for long periods of time (between one and six months) [6].

The heterogeneity of BC results from a diversity of factors, in general, dominated by the morphological characteristics of tumors and the origin of the neoplasms [7]. The challenge of automated detection and/or classification of breast tumors arises from their variety of types and subtypes. Magnetic Resonance Imaging (MRI) assessment is a more time-consuming task. When compared to Mammography (MG), the workload of radiologists/physicians increases, as the efficiency of this modality is affected not only by the variety of morphological characteristics of each specific tumor phenotype and its origin, but also by the human fatigue of having to read/review hundreds of slices (images) to analyze each individual patient case. Therefore, the detection of BC pathological lesions is still an unsolved problem with these added difficulties for radiologists in terms of MRI analysis.

In the last two decades, clinical trials have been reporting the utility of breast MRI in detecting cancers not apparent by MG or clinical examination [8,9,10]. Currently, the problem of BC detection has been addressed by applying Artificial Intelligence (AI) techniques, namely Machine Learning (ML)/Deep Learning (DL) and Computer Vision (CV) algorithms and methods. In this sense, Faster R-CNN models has been primarily used for general object detection tasks and more recently in BC detection tasks. As main advantages, Faster R-CNN-based models have demonstrated high accuracy to detect of breast cancer-related pathological lesions in several medical imaging modalities (e.g., MG, MRI, Ultrasound). Faster R-CNN efficiently generates region proposals, which could help in identifying potential regions of interest in MRI and can be adapted for multi-class detection settings, making it suitable for spotting different phenotypes of pathological lesions in MRI-based BC detection tasks.

This work has focused on answering the following research question: is it possible to improve the detection of pathological BC lesions (i.e., BC phenotypes) in MRI by using Faster R-CNN-based detection models? To solve this research question we had to address three issues/problems: (1) performing a research and requirement analysis work to select a dataset to serve as a golden standard (representative of the main subtypes of BC), with sufficient data, i.e., digital content (annotated MRI images) and associated metadata (genomics data); (2) developing an innovative method for patient’s images (slices) selection; and (3) fine-tuning, training and testing several R-CNN models.

### 1.1. State of the Art

#### 1.1.1. Medical Imaging Modalities

In general, most of the work developed to date on Computer-Aided Detection/Diagnosis (CADe/CADx) methods/systems in BC pathological lesions (tumors), particularly on classification systems, is based on Histopathology (HP) biopsy images and or bi-dimensional (2D) X-ray MG. However, other modalities including Ultrasound (US), Digital Tomosynthesis (DT), Computer Tomography (CT), and MRI have been explored [11].

Of all BC tumors, 70% to 80% are related to one of the two major histopathological types: Invasive Ductal Carcinoma (IDC) and/or Invasive Lobular Carcinoma (ILC). These two main classes group most of the diverse spectrum of above mentioned types [7,12]. The remaining 30% to 20% of BC tumors not typed as IDC or ILC fit into other categories weakly represented in datasets, a characteristic that traditionally is an impairing factor for DL solutions.

Histopathological biopsy is an invasive and conclusive medical imaging modality, in which the details of the phenotype of the pathological lesion can be better observed/identified [13]. Although their results in terms of ML algorithms for multi-class classification still are not optimal [14], the efforts/works in this direction are currently being intensified [15].

MG is one of the most used modalities for early screening. This modality is not only useful to help determine breast masses (nodules), but also their location [15]. Nevertheless, along-side 2D MG additional screening with supporting modalities, such as the US, is required as an MG has low sensitivity, particularly in presence of dense (fatty) breast tissue images [16].

The advantage of US, compared to other more invasive techniques, is the fact that patients do not receive ionizing radiation. However, it has some limiting factors. For instance, the US is limited in its ability to distinguish between calcifications and cancerous masses. Its poor image quality is prone to cases of misinterpretation; in part, US is also used as an auxiliary methodology in MG screening and to aid in decision making tasks, for example, by prescribing additional exams with other modalities, such as biopsy tests [17].

Since it was introduced more recently, DT is one of the less studied modalities and there are less available public data, i.e., annotated datasets released for public domain [18]. Compared to 2D MG, some recent published works have indicated that DT allows for more effective BC diagnostic capability and produces more reliable interpretations. Its 3D views prove to be more powerful for the detection of abnormalities [19]. However, as it is more challenging to handle 3D data for automated detection, 2D MG classification results were demonstrated to be more efficient [20].

MRI is the most accurate radiological method for accessing tumor size, multifocality, and multicentricity, it has better sensitivity and higher diagnostic accuracy [21], but it’s still not introduced/used for screening on its own, due to it is considered too expensive and time-consuming [22] as for the analysis and assessment of MRI sequences, which can easily group several hundreds of images for a single patient. Until a few years ago, MRI had only been used as an auxiliary modality when the combination of modalities, such as MG and US, did not provide conclusive results with the aim of increasing detection rates. In addition, MRI was predominantly used to evaluate other features, such as size and identification/detection of other tumor areas [16]. As aforementioned mentioned, MRI, compared to other imaging modalities, such as MG, is particularly expensive and requires a higher physician workload to evaluate patient cases, nevertheless, the same author states that 7 out 8 of his studies reveal that the mean sensitivity of MRI is 95.6%. Therefore, it is crucial to reduce human effort in reading/evaluating MRIs and take advantage of its high sensitivity performance/capacity to detect pathological lesions. The studies reviewed address both detection and classification—as one of the two main types—the binary early detection (including classification), between benign and malignant tumors, or the multiclass classification, which aims not only to distinguish benign and malignant but also different tumor types and subtypes/phenotypes.

#### 1.1.2. Artificial Intelligence/Machine Learning/Deep Learning Methods

Artificial intelligence (AI), ML and DL are popular terms sometimes used interchangeably, particularly when companies are marketing their products. The terms, however, are not synonymous; there are important distinctions. AI refers to the simulation of human intelligence by machines. ML as a branch (type) of artificial intelligence that uses algorithms to learn from data to make sense of it or predict a pattern. ML uses algorithms and methods to find hidden insights within data without being programmed where to look or what to conclude. Based on this, machines are trained and “learns how to perform a job” by analyzing relevant data, allowing it to understand how to accomplish the task and then evolve its performance. DL is a branch (evolution) of ML supported on Artificial Neural Networks (ANN), which uses advanced computer programming and training to understand complex patterns hidden in large datasets. The ability of DL algorithms to process massive amounts of data simultaneously and perform analyses quickly makes this approach highly scalable.

The ANN comprise a set of algorithms that enable computers to learn patterns from large volumes of data. Initially, inspired on the functioning of the human brain, it consists of an acyclic directed graph of neurons (nodes) organized in layers which are capable of holding a state and, are updatable by using a backpropagation approach. They are composed of several layers: an input layer, an output layer and at least one hidden layer. The input layer has as many nodes as the number of data points of each input data instance, e.g., a numerical representation for every char in a string. The output layer has as many nodes as the number of classes the model should be able to predict. As for the hidden layers, the purpose of each of its nodes is to calculate the normalized value of the weighted sum of the inputs multiplied by the node current state weight. These calculations make ANNs very expensive computationally and back in 1943, when they were first studied by McCulloch & Pitts [23], computers were way to limited, which made them inadequate for processing massive amounts of data. Today machines are way more powerful when compared with the few existing ones in the first half of the XX century. Still ANN algorithms with multiple hidden layers are very demanding computationally.

These ANN with multiple hidden layers are also known as DL. DL algorithms are a specialization of ANNs. Convolutional Neural Networks (CNN) are a subset of DL algorithms used for image’s classification. In general CNNs consist in a deep ANN preceded by a variable range of layers intended to reduce the amount of data points (pixels) of each data instance (images). This process is also a way of feature selection/extraction. A single MRI image with 512 × 512 pixel has 262,144 data points, this is where feature extraction becomes relevant.

Detection is essentially a discovery process. A process capable of analyzing either the presence or absence of something or at the more generic level of computational language, of objects. Detection is an important process of CV, and a preliminary process for other processes such as segmentation and classification [24].

The Textural Analysis (TA) of MRI is already identified as having the potential to assist in classifying tumors as benign or malign [25,26]. There are also studies on DL algorithms for other modalities such as MRI Background Parenchymal Enhancement (BPE) classification, which is not exactly a BC classification approach, but may affect diagnostic accuracy becomes relevant for this study. Borkowski, K. et al. implemented two well-performed CNN models for the classification of BPE to categorize its in four classes/groups: minimal, mild, moderate, and marked [27]. Breast tumors are divided into several categories, 20 major types, and 18 minor subtypes. Apart from the binary classification, over 70% of the BCs belong to one of two types of BC, Invasive Ductal Carcinoma (IDC) and Invasive Lobular Carcinoma (ILC) [12]. This uneven distribution of tumors results in added difficulty in automatically detecting BC using machine (deep) learning techniques. Breast tumor type distribution alone embodies some concerns in terms of dataset representativeness. As an example, in terms of training ML models the standard is to split between training and testing subsets—previously randomized—with percentages between 70%–30% to 85%–15% respectively, depending on several aspects. This approach enables testing the model in fresh data that was previously isolated from the training data subset, such as, the size and number of features of the dataset. Moreover, in terms of DL models, the dataset is normally split into 90% for the training set and 10% for the testing set. However, as DL algorithms are used when there are available massive amounts of data, this ratio is used when we have Bigdata. In terms of cancer tumors, with less than 30% representing 18 major types of BC, it means that—assuming a dataset with all tumor types—the training set eventually may not include some of BC types, and, probably, the testing set would not include some of the tumor’s types considered into the training phase.

### 1.2. Contributions of This Work

As will be explained in detail in the following sections, we propose a novel Faster R-CNN-based framework to automate the detection of BC pathological Lesions in MRI. As a main contribution, we have developed and experimentally (statistically) validated an innovative method improving the “breast MRI preprocessing phase” to select the patient’s slices (images) and associated bounding boxes representing pathological lesions. In this way, it is possible to create a more robust training (benchmarking) dataset to feed DL models, reducing the computation time and the dimension of the dataset, and more importantly, to identify with high accuracy the specific regions (bounding boxes) for each of the patient’s images, in which a possible pathological lesion (tumor) has been identified. As a result, in an experimental setting using a fully annotated dataset (released to the public domain) comprising a total of 922 MRI-based BC patient cases, we have achieved, as the most accurate trained model, an accuracy rate of 97.83%, and subsequently, applying a ten-fold cross-validation method, a mean accuracy on the trained models of 94.46% and an associated standard deviation of 2.43%.

## 2. Dataset

**Figure 1:**
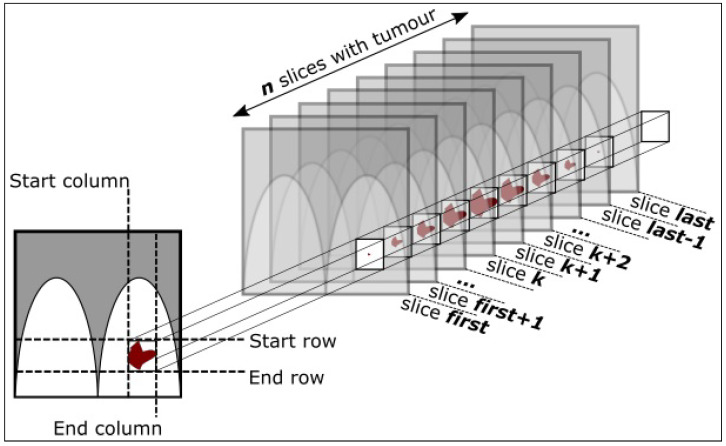
Illustration of the annotation schema for the bounding boxes delimiting the tumor. The first and last few slices may have loose bounding boxes (effect produced by lesions edges on the 3D boxes).

This work aims to explore and validate algorithms and methods for supporting the detection of BC pathological lesions in MRI-based patients’ cases. In these sense, after visiting/reviewing several datasets released for the public domain, we determined that the dataset with the best characteristics/conditions/criteria to develop our work is the Duke-Breast-Cancer-MRI dataset (DukeBC). DukeBC comprises a high quality compilation of dynamic contrast-enhanced MRI of BC patients cases with tumors locations and associated metadata (i.e., genomics data). This dataset comprises a collection of 922 positively diagnosed (biopsy proven) BC patients’ cases fully annotated and anonymized captured/collected by the Duke Hospital, Durham, North Carolina, USA. DukeBC encompasses a large number of visual traits capable of distinguishing different BC phenotypes, which made it ideal for developing and testing CV algorithms and DL models to support BC cancer lesions detection methods. The patient’s age range is 21 to 89 years old, and the average age is 52 years old. Annotations were performed by 8 radiologists to whom the cases were randomly assigned. The pathological lesions (tumors) annotations are identified using 3D bounding boxes delimited by 2D coordinates, plus a set of slices where the tumors were found, as depicted in Figure 1. For each patient are available 5 or 6 image sequences (series). A sequence is a series of radio-frequency pulses, each one with its specific settings, resulting in a set of images (slices). Some patients’ cases present MRI sequences without fat suppression, however, these sequences were not used as they do not have (match) the corresponding annotation [28].

The MRI scans were performed using equipment from 2 manufacturers, GE Medical Systems and Siemens, 8 distinct models were used, as illustrated in Table 1.

Overall, the dataset is composed of a total of 772,439 images (slices) distributed by 922 patients, an average of 838 images per patient. The complete dataset occupies a disk storage space of approximately 342 gigabytes, with each DICOM image averaging 443 kilobytes. All images are square in shape and the size varies between 320 × 320 pixels (33 cases), 448 × 448 pixels (261 cases), and 512 × 512 pixels (628 cases). Nevertheless, the size of the images remains the same in all images from the same patient (patient case).

It can be observed that some relevant quantitative features of the pathological lesions, i.e., shape and size differ highly, depending on the tumor type and subtype. Besides, it is important to note that the minimum number of slices in which a tumor is identified/detected in a patient case is 2 slices and the maximum number is 131 slices. A limitation (restriction) observed in this dataset is the fact that, even if more than one lesion were detected, only the largest pathological lesion (tumor) was annotated for each patient case, i.e., physicians/radiologists only annotated one (the largest) biopsy-proven tumor for each patient case [28]. This can be a cause of undesired False Positives (FP) occurrences (Type I Error), thus, it’s expected to have mismatched detections. Without ground truth annotations it is impossible to validate these FPs. As the dataset doesn’t include patients’ cases without BC pathological lesions, it is only possible to measure True Positives (TP) and False Negatives (FN) patient-wise, in which we can compute patient-wise metrics. Thus, there is no means to assess whether the FP are real or just a result of the lack of annotation. This inability to assess correctly the tumors detected (FP and FN) prevents to compute some significant metrics, such as, accuracy and recall as a result. To have all the tumors annotated would undoubtedly be an added value in the validation of the model.

## 3. Proposed Framework

**Figure 2:**
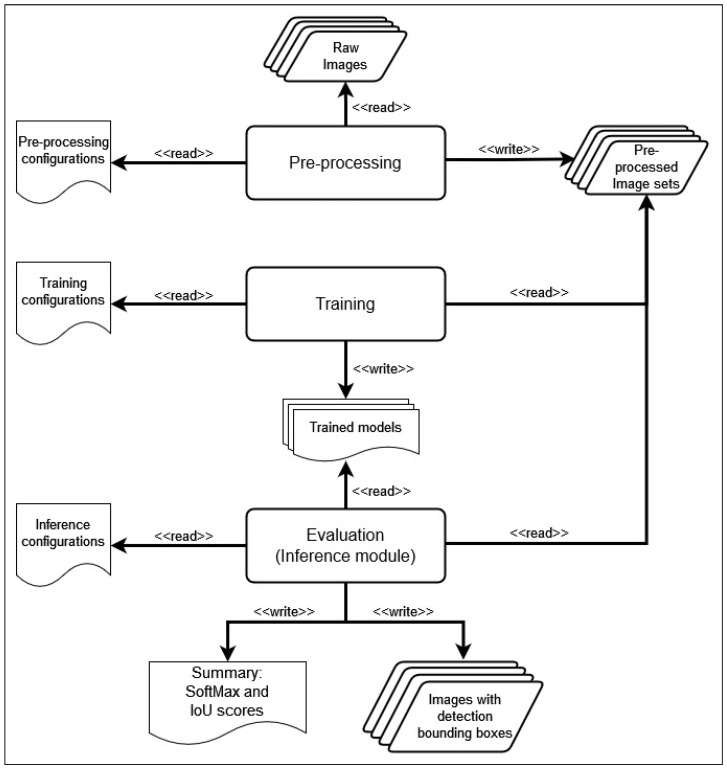
High-level workflow of the proposed method.

Figure 2 shows the high-level workflow of the proposed framework comprising three well-defined phases: preprocessing, training, and evaluation, being the main contributions in this work associated to the preprocessing phase.

The focal point of this work is to help physicians detect BC. In this sense, we have trained CNN models for learning pathological lesions (tumors) patterns using a fully wide-ranging annotated (golden standard) dataset. Our main goal is to improve the automatic detection of BC pathological lesions (BC phenotypes) in MRI, i.e., new (unannotated) patients’ cases.

For that purpose we have trained models using a TensorFlow variant of the Faster R-CNN Inception ResNet V2 method/architecture [29,30,31]. Specifically, we aim to point out where—sequence and slice—the tumor is most noticeable as well as its classification score. The most negative weakness—or damaging aspect—of the medical assisting AI model are the FN errors (Type II Error) which can produce misleading evaluations, i.e., not identifying a tumor when it exists. The tumors potentially detected by the model should indicate to radiologists which sequences and in which slices they can perform further research/analysis.

## 4. Preprocessing

This step aims to prepare the dataset before starting the training process. It includes loading, selecting, extracting, and preprocessing the necessary information from the DICOM files (i.e., digital content—MRI images and associated metadata). This procedure is crucial as DICOM image metadata includes specific information and relevant features about the scanning options used and other annotations.

For resolution (size) normalization issues, the images of all patient cases were resized/ converted to a resolution of 448 × 448 pixels. Nevertheless, the original size is not discarded given that it is necessary to calculate the coordinates of the bounding boxes for the new image size. Next, with all the necessary data/information, well identified and organized, for each image a XML annotation file is generated. This file includes the coordinates of the bounding boxes for each lesion as well as the object tag (tumor). Also, to increase image diversification, several operations of data augmentation are performed. This procedure typically covers specific transformations for inverting, rotating, adjusting brightness and images contrast. This procedure is described in more detail in the next section, as it is carried out in the DL model.

To be ready for the training process, patients’ cases are separated into train and test sets. It is mandatory that the splitting process be performed by patient, rather than by image, to ensure that images from the same patients are not included in both datasets (training and test). The goal of choosing only one set of images from each patient is to exclude images that have too little information or too much noise. The splitting process is only performed after the dataset is split into train and test subsets (patient-wise).

To avoid problems of extreme looseness in the bounding boxes, i.e., to not include slices where lesions represent very small areas of the bounding boxes, we filter/select which tumor slices should or should not be included in the training set. It is important to note that the patient cases selected to be part of the test subset must include all slices (images), i.e., the inference process does not take into account the bounding boxes annotations. Therefore, the trained model is expected to be able to detect all possible tumors present in each patient case. Reiterating the matter, the loose bounding box is only a problem for the training process since it is desirable not to introduce noise into the model.

List 1: Image preprocessing steps.

- Load images identifiers to memory and DICOM identifiers
- Filter images by scan options (annotation are only applicable to fat supressed images)
- Transform images to fixed shape and size
- Generate annotation XML file for each image
- Generate dataset split declaration file
- Apply slice selection algorithm
- Copy images and annotations to final directory

### Patient Slice Selection Method

Our method focuses on the identification/selection of a reduced set of slices (images) for each patient case, with better features to facilitate the detection of breast tumors. This process is performed by a set of scripts that generates the training artifacts fed to the TensorFlow Object Detection API. On one hand, the dataset has complex preprocessing requirements, due to the specifications described in Section 2. On the other hand, the dataset is great in size, over 340 GB, which takes a lot of time to process and can result in memory shortage if handled incorrectly.

**Figure 3:**
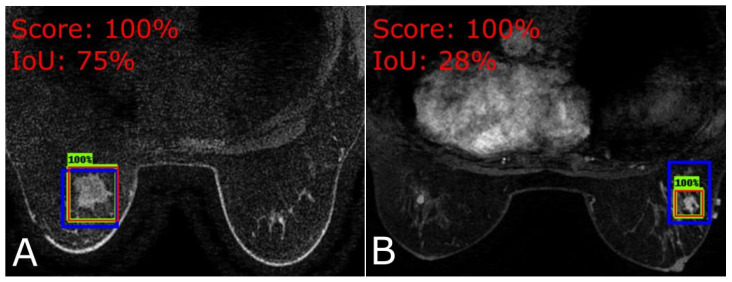
Blue box: ground truth; green box: detection; red box: true positive. (**A**) Slice from patient 45 denoting a tight fit to the tumor. (**B**) Slice from patient 640 denoting a loose fit to the tumor.

Most of the patients in this dataset have tumor slices that are not tightly fit into the bounding box, as depicted in Figure 3 and previously shown as well in Figure 1. In Figure 1, the initial and last slices (images) of the patient show significantly small/loose tumors within the bounding boxes. This can lead to the decline of the model performance as it introduces noise that will be back-propagated to the network (model) weights ([32]).

Preliminary tests with both, all slices and too few slices, revealed difficulties in the model’s pattern learning. Removing slices means removing information. We need to avoid both removing too many slices, which could lead to loss of important data, and removing too few slices, which would include excessive noise from loose boxes. To address this problem, a specific purpose-built function was developed to calculate the appropriate number of slices for removal.

By visual analysis of the dataset, in most patient cases, we think that it would be appropriate to remove one half of the slices. However, given that there are many smaller tumors with a total number of slices between 2 and 10, it would not be a good approach to remove one half of these images. To address this issue, we developed a logarithmic funneling function (see Equation (1)) to dynamically determine the amount of the slices to keep and the amount of slices to be removed. Of course, with such a configuration to prevent the over-removal of slices in those smaller tumors.

**Figure 4:**
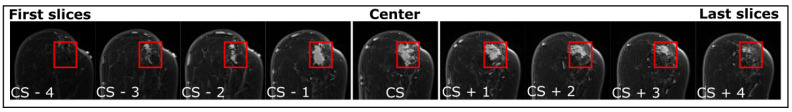
Illustration of bounding boxes looseness on a 9-slice tumor. Slice CS represents the central slice. CS-4 represents the first slice and CS+4 represents the last slice. The central slice (CS) is well fit to the bounding box, from CS to each of the sides, the bounding boxes have increasing looseness.

The concept behind the methodology for excluding the slices with loose bounding boxes, illustrated in the tumor represented in Figure 4, is to exclude slices CS-4, CS-3, CS+3, and CS+4. Slice CS-2 and CS+2 already introduce noise, but show some beneficial info, and the bounding boxes are not as loose as the end slices. As mentioned, the slice selection process is only applied to the train dataset. The goal is to reduce the number of images where the tumor is not fit into the bounding box in order to reduce the noise surrounding the lesions. By applying Algorithms 1 and 2 to a sample patient case containing 9 slices, we end up removing 4 slices and keeping 5 slices, as it is shown in Figure 4. As for the test dataset, all slices are used to increase the chance of detecting the tumor.

In Equation (1), *a* and *b* are constant values to control the funneling effect. The values of *a* and *b* were heuristically obtained through trials, starting with *a* set to 1 and *b* set to 2. The value computed from this equation is then rounded up, and the result obtained is considered the total number of slices to be maintained/selected for a given patient. Finally, it was found that using the values a=0.75 and b=1.9625 respectively led to satisfactory results. We do not assume that these are the best values possible for *a* and *b*. These are the values that shaped the funneling effect in a way that we considered suitable to achieve good results. However, we do not rule out the possibility that better results may be obtained with different values.

By using the aforementioned settings, a patient with a tumor ranging 108 slices, would keep only 55 central slices after applying this equation. A total of 53 slices would be removed, 26 slices from the beginning of the tumor and 27 slices from the end of the tumor. To compute the total number of slices to be removed, the value resulting from Equation (1) is subtracted from the total number of tumor-containing slices for that patient. The resulting value is divided by two to determine the number of slices to be removed from each side (left and right) in each specific patient case. If the result is not an integer value, the value is rounded down for the initial slices to be removed and rounded up for the final slices to be removed.

(1) f(x)=total_number_of_slices_with_tumor∗a∗ln(b)

**Algorithm 1** Pseudo-code: Application of the funneling function. As the result of the funneling function is a real number, the value must be converted to an integer. We do so by rounding the value up (ceiling).
1:**function** CalcNumOfSlicesToRemove(  total_number_of_slices_with_tumor, total_number_of_slices  )2:    3:  Real funnel_result:=funneling_function (total_number_of_slices_with_tumor)4:    5:  **return** (Integer) total_number_of_slices−ceil (funnel_result)6:
**end function**



**Algorithm 2** Pseudo-code: Based on the total number of slices, this algorithm calculates the number of slices to remove from each end of the slices with tumor range. When the total number of slices to remove is an odd number, we round down (floor) the number of slices to remove from the beginning of the range and round up (ceil) the number of slices to remove from the end of the range.
1:**function** NumberOfSlicesToRemoveFromEachSide(  total_number_of_slices_with_tumor, total_number_of_slices  )2:    3:  Integer number_of_slices_to_remove:=CalcNumOfSlicesToRemove(   total_number_of_slices_with_tumor, total_number_of_slices   )4:    5:  Real number_of_slices_to_remove_on_each_side:=num_of_slices_to_remove/26:    7:  **return** (Tuple<Integer, Integer>) (   floor(number_of_slices_to_remove_on_each_side),   ceil(number_of_slices_to_remove_on_each_side)   )8:
**end function**



Results of the application of the Algorithms 1 and 2 are illustrated in Table 2. In the descending gradient can be observed that gradually the total number of slices is decreasing when compared to the original count. In the minimal case—patients with tumors with a maximum count of 2—no slice will be removed by the algorithm. As the total number of slices increases, the number of slices to be removed also increases. This is also observable in the ascending gradients representing the number of slices to remove at the beginning and end of the tumor.

A contribution of the proposed framework lies in the fact that it allows to extract/ consider only those slices (images) in which a lesion (or a part of a lesion) is sufficiently represented within the bounding box selected by the physician, i.e., the percentage of pixels of the lesion occupies a significant area that allows its use to differentiate/detect the lesion. Table 3 shows the concentration of patient cases (PC—Patient Count) grouped according to the total number of slices (SC—Slice Count). The SC ranges between 2 and 131, and most of the patients have SC ranging between 10 and 30 slices. For instance, on a patient with a 9 slices tumor, as illustrated in Figure 4, we would get to keep only 5 slices (middle slices) and would remove 2 from each side. It is noticeable in the figure that the tumor bounding boxes are loose in both extremities having more than half of area without tumor representation.

## 5. Training

### 5.1. Environment Setup

The experiments consisted in two step, two preliminary training’s and a 10 fold cross-validation using all in the same environment as described in Table 4. As mentioned we use a Faster R-CNN network running on Ubuntu 20.04. All scripts were developed using Python 3.9.12 and the Python’s virtual environment was managed with miniconda 4.12.0.

### 5.2. Model Parameterization

#### 5.2.1. Epoch Configuration

One epoch is one network pass through the data. One step is one network passing through one batch of images, backpropagation included. Using TensorFlow Object Detection API, the model and network are configurable by editing a settings file called pipeline.config. It has settings for all stages of the Faster R-CNN workflow. The network has parameters to define the total number of. Batch size, which is a setting highly related to the GPU memory available, is shown in Table 5. The batch size must be divisible by the number of GPUs used. In this project, 4 GPUs were used and a batch size of 32, which means a batch of 8 for each GPU and, 5 GB of GPU memory allocated to each image. The number of epochs is a relation between batch size and the total number of steps, as demonstrated in Equation (2).

(2) $$f\left(x\right)=\frac{total\_number\_of\_steps*batch\_size}{train\_set\_size}$$

#### 5.2.2. Learning Rate

The learning rate is one of the most important parameters, it controls the magnitude of the values used in back-propagation to update the weight of each node. A smaller learning rate will cause the model to take longer time to train. On the other hand, a higher learning rate will train faster, but it may never reach the optimal value of the weights. This is because in this scenario the weights would be constantly updated with values higher than the difference between the current value and ideal value, and thus may never converge to an optimal value or at least to point near the optimal value.

**Figure 5:**
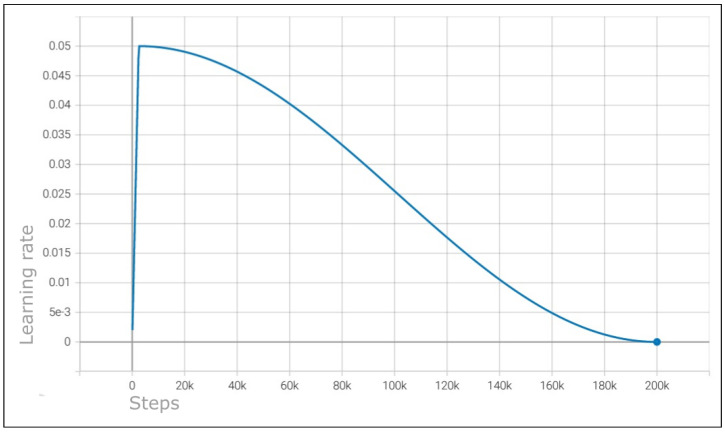
0.05 learning rate cosine decay visualization on a 200,000 steps trained model.

In this project we have used a cosine learning rate decay approach. The goal is to initially use larger values to update the weights, but as the model trains, the value will gradually decrease. This leads to a higher probability of finding the optimal value and thus avoiding the above described effect. In Figure 5, we shown an example of the decay effect using the cosine learning rate decay in a 200-thousand-step trained model.

#### 5.2.3. Data Augmentation

The model was configured to apply the following data augmentation transformations randomly: 50% chance to flip the image horizontally, 50% chance to flip the image vertically, 50% chance to make a 90° rotation, changes image brightness up to a maximum delta of 0.05, and image contrast modification by a value between 0.5 and 0.95. The value is to be multiplied by the image’s initial contrast. This process, along with a high number of epochs, resulted in the data diversifying and expanding in number.

#### 5.2.4. Network Weights Initialization

The network weights were initialized with values from a uniform distribution within −limit and +limit. Being the limit calculated as demonstrated in Equation (3).

(3) $$limit=\sqrt{\frac{3}{n}}$$

#### 5.2.5. Classification (Scoring)

The network will be configured to use the SoftMax score converter on the last layer to output probabilistic value because of the output nodes.

### 5.3. Preliminary Model Training

#### Preliminary Models Setup

The preliminary models (hereinafter referred to as model A and model B) were carried out using the dataset setups, as described in Table 6. In model A we used all slices available. In model B we used the proposed slice selection method described in Pre-Processing Section 4. Both setups share the same patients’ cases for training (866) and test (56) datasets. The test dataset is the same for both models, as no slices are excluded for the test set and case selection is equal. Model A counts a total of 77,963 images for the training set and Model B, which used the proposed slice selection method, has 53% of model A images count for training, i.e., 41,317 images. The training process ran 160 epochs on batch sizes of 32 with a learning rate of 0.05 and cosine decay.

### 5.4. Cross-Validation

#### Cross-Validation Setup

The model is trained using a k-Fold Cross-Validation method with k = 10. According to Marcot et al. [33] using k = 5 folds is usually sufficient for large datasets (e.g., 5000 data instances) while for smaller datasets, a higher k values of folds can be beneficial. Although the dataset used has a large number of files, i.e., slices (images), due to the fact that we have chosen a patient-wise approach, the collection has a total of 922 data instances (patients’ cases), which, in turn, means a significantly reduced size. Using 5-fold cross-validation would result in 5 training sets with 80% of the whole dataset (737 patient cases). By using 10-fold cross-validation we can count with 10 training sets with 90% of the whole dataset (829 patient cases) each fold. Moreover, it enables a finer estimation of the classifier’s performance. Thus, using k = 10 folds, 10 models were trained on different combinations of the training and test sets. To produce this partition the patients are shuffled and divided into 10 batches. The training set will always have 9 batches and the test set 1 batch. For each of the 10 training phases, a different batch (1/10 of the patients) is used as the test set.

**Figure 6:**
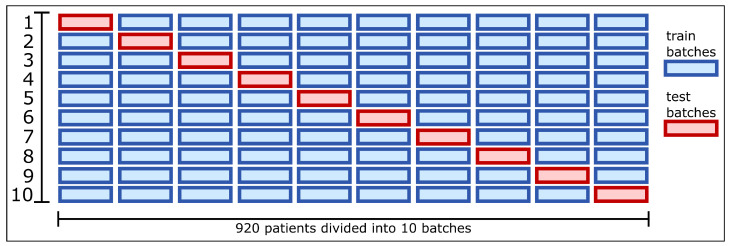
10-Fold cross-validation scheme.

At the end of all 10 training phases, the predicted output scores and bounding boxes are used to calculate the evaluation metrics. Thus, in each training phase, the model is trained on 90% of the total patients and tested on 10% of the total patients. The 10-fold cross-validation scheme is depicted in Figure 6. A particular caveat regarding this method is that the dataset has 922 patients. As 10% of 922 is 92.2 and the rest of the patients are not divisible by 10, the 2 extra patients will be used in the training sets.

The specific slices to be used in the training sets are selected according to the procedures described in Section 4. As mentioned in the same section, the slice selection algorithm is only applied to the training set as it is advantageous to test the model on all slices of each patient to properly evaluate the model.

The experiment was conducted on the Dukes’ Hospital Breast Cancer MRI Dataset described in Section 2. As referred, we split the dataset patient-wise, and only after patient splitting process the slice selection process starts. As forementioned, each tumor has its particular shape and size, and, of course, a different number of slices where the tumor is identified. This means that each of the cross-validation folds has a different total image count, as depicted in Table 7. Nevertheless, the network parameterization was the same for all folds. The training process ran 162 epochs on 200,000 steps, with a batch size of 32, a learning rate of 0.05, and cosine decay.

Because the cross-validation train set has less patients, and therefore less images, although we used in cross-validation the same hyper parameters as the previous section model B the number of epochs increased accordingly.

## 6. Evaluation and Discussion

### 6.1. Preliminary Results

These results allow us to evaluate and draw some conclusions about the effect of the slice selection algorithm as opposed to using all images. As summarized in Table 8, Model B achieved better results in all metrics. Not only Model B has fewer FN (patient-wise), a better SoftMax score, and better Intersection over Union, but also it has trained in fewer images (53% of total slices of Model A) and therefore, for the same number of epochs, the Model B is faster to train taking less 27.16 h than Model B.

### 6.2. Cross-Validation Results

Table 9 summarizes the results standard deviation calculus. Across all folds, the average accuracy in detecting the BC tumor is 94.46% with a standard deviation of 2.43%, which is a good result. The average IoU and the average score are important results to evaluate the models and their performance object-wise. But, as for the ability to assist radiologists in prescriptive analysis, they are of minimal value.

The average IoU and scores are relatively low, reflecting the failure to detect several slices, generally corresponding to the edge slices. More important are the maximum metrics which generally score well, with 89.16% for the average maximum score and 69.08% for the average maximum IoU in cases where the tumor was detected. These two metrics effectively reveal the model’s ability to detect and pinpoint the slices where a tumor is best identifiable, thus providing actionable insight and aiding physicians in prescriptive analysis.

The best performing folds were the 1st and the 3rd folds. The 3rd fold is for best accuracy (97.83%) and Average Score @ Max IoU (91.56%); the 1st fold is for best Average Max IoU (71.34%).

### 6.3. Model Comparison

Since our method presents a patient-wise rather than image-wise perspective for pathological lesions (tumors) detection, direct comparison with results obtained by other authors has been a challenge. With exception to [34] we could not find other articles that use the same metrics and at the same time focus on patient rather than on each image. However, our method achieves promising results in terms of overall accuracy, demonstrating its potential to detect BC tumors more comprehensively. The lack of comparable studies using similar metrics and patient-wise analysis highlights the novelty of our approach. However, we provide an overview of other authors’ results in Table 10. The prospective impact of our methodology goes beyond object detection, focusing on the patient as a whole has the potential to aid in BC screening planning by pin pointing suspicious cancerous lesions.

## 7. Conclusions

In this work we have developed an innovative methodology for the preprocessing of patients’ cases including magnetic resonance imaging (MRI) that allows the selection of a reduced interval of slices (images), in which there are annotated (automatically or manually) bounding boxes representative of previously identified pathological lesions. The proposed approach allows (i) minimizing background noise by selecting only slices (images) that include bounding boxes with a sufficient area (representative percentage) of identified/annotated pathological lesions (ii) reducing the number of slices per patient needed to build reference (benchmarking) datasets and thus, (iii) reducing the computational time necessary to train ML/DL detection models. Based on this, and in order to answer the research question that motivated this work, we developed a full lifecycle framework for training and testing “Faster R-CNN-based deep learning models”. The proposed framework has been successfully validated, in an experimental setting, using an annotated dataset (Duke-Breast-Cancer-MRI dataset - DukeBC) released for public domain. DukeBC comprising a high quality compilation of dynamic contrast-enhanced MRI of 922 BC patients cases with tumors locations and associated metadata (i.e., genomics data). Trained Faster R-CNN models for detecting pathological lesions were extensively validated, using a 10-fold cross-validation technique, achieving a satisfactory mean accuracy of 94.46% with a standard deviation of 2.43%. Therefore, although it is difficult to make a fair comparison with other previously published works, because in some cases the authors used other datasets (with fewer patient cases), or in other cases the analysis/validation performed is image-wise and we use a patient-wise approach, we demonstrated that based on the proposed framework it is possible to improve the detection of pathological BC lesions (i.e., BC phenotypes) in MRI by using Faster R-CNN-based detection models.

An identified drawback is the fact that parameters used to fed the funneling function (Algorithm 1) were heuristically defined and although we consider the results obtained satisfactory, we cannot guarantee that this is the best configuration (i.e., selected parameters). Therefore, we recognize it is needed more experimentation. Finally, we identified as the main limitation to perform this work the training time, since finding the optimal arguments for the funneling function took us several months.

Future work will focus on both expanding the MRI-based dataset by including new fully annotated patient cases and improving the accuracy of DL methods for the detection and classification of pathological lesions (i.e., phenotypes) of breast cancer.

## Figures and Tables

**Table 1:** Case count by MRI equipment model and manufacturer.

Manufacturer	Model	Cases
GE MEdical Systems	Optima MR450z	98
	Signa Excite	10
	Signa HDx	272
	Signa HDxt	248
Siemens	Avanto	179
	Skyra	57
	Trio	1
	Trio-trim	57

**Table 2:** Calculated values by applying the funneling function on Equation (1). Some rows are skipped to save vertical space. (Rows with three dots represent a sets of omitted rows).

Patients Total Number of Slices	Patient Total Number of Slices to Remove	Number of Slices to Remove from Start	Number of Slices to Remove from End	Number of Slices to Maintain
2	0	0	0	2
3	1	0	1	2
4	1	0	1	3
5	2	1	1	3
6	2	1	1	4
7	3	1	2	4
8	3	1	2	5
9	4	2	2	5
10	4	2	2	6
…				
20	9	4	5	11
…				
30	14	7	7	16
…				
40	19	9	10	21
…				
50	24	12	12	26
…				
60	29	14	15	31
…				
70	34	17	17	36
…				
80	39	19	20	41
…				
102	50	25	25	52
108	53	26	27	55
111	54	27	27	57
119	58	29	29	61
131	64	32	32	67

**Table 3:** Count of the number of patients PC with the total number of slices SC.

(2–16)		(17–31)		(32–46)		(47–62)		(63–77)		(79–131)	
SC	PC	SC	PC	SC	PC	SC	PC	SC	PC	SC	PC
2	1	17	34	32	17	47	6	63	3	79	1
3	4	18	39	33	12	48	3	64	3	80	1
4	8	19	26	34	11	49	6	65	3	81	3
5	3	20	26	35	12	50	4	66	3	82	4
6	10	21	30	36	4	51	7	67	1	85	1
7	15	22	32	37	9	52	4	68	1	89	1
8	19	23	19	38	10	53	4	69	5	90	1
9	27	24	23	39	7	54	4	70	1	95	1
10	24	25	24	40	4	55	3	71	1	96	1
11	38	26	21	41	4	56	4	72	1	97	1
12	43	27	23	42	7	57	3	73	1	102	1
13	44	28	18	43	8	58	1	74	2	108	2
14	35	29	14	44	6	59	2	75	1	111	1
15	36	30	8	45	4	60	1	76	1	119	1
16	33	31	17	46	10	62	2	77	1	131	1

**Table 4:** The systems consists of two super computers, each having these specifications with CPUs. This cluster belongs to the VISTA Lab Research Center of the University of Evora, Portugal.

Cluster—Vision at VISTA Lab (2x)		
CPU	Designation	Dual AMD Rome 7742
	Cores	128
System memory	GB	1000
GPUs	Designation	NVIDIA A100 Tensor Core GPU
	N. of GPUs	8
	Cores	6912 (total 55,296)
	Memory (GB)	40 (total 320)

**Table 5:** System configuration and benchmark.

	Cluster Configuration		Benchmark	
GPUs used	Total GPU memory (GB)	Batch size	Steps per second (avg)	Images processed per second (avg)
4	160	32	1.90	60.8

**Table 6:** Dataset setup for preliminary models. The train set image count for model A is 189% of the size of the model B train set. The train set image count for model B is 53% the size of model A train set.

Designation	Slice Selection Method	Image Count	
		Train set	Test set
Model A	All slices with tumor	77,963	Same test set with 5031 images
Model B	Funneling method described in Pre-processing section	41,317	

**Table 7:** Cross-validation of patient and image count for each fold.

Fold	1	2	3	4	5	6	7	8	9	10	Avg
Total image	39,903	39,844	39,515	39,220	39,821	39,347	39,159	39,791	39,551	39,773	39,592
Images per patient (avg)	48	48	48	47	48	47	47	48	48	48	48

**Table 8:** Summarization of the preliminary results.

Metric	Model A	Model B	Std Dev
Train time (hours)	56.65	29.34	±19.315
True Positives cases	53	54	±0.707
False Negatives cases	3	2	±0.707
False Negative slices	2611	2212	±282.136
Accuracy	94.64%	96.43%	±1.27%
Average IoU	24.63%	31.58%	±4.91%
Average Score	48.78%	59.05%	±7.26%
Average Max IoU	59.61%	69.40%	±6.92%
Average Score @ Max IoU	81.61%	91.30%	±6.85%

**Table 9:** Cross-validation results and standard deviation calculation for each fold.

Fold		1	2	3	4	5	6	7	8	9	10	Avg	Std Dev
False Negatives		3	4	2	6	3	5	6	8	5	9	5.10	±2.23
Accuracy (%)		96.74	95.65	**97.83**	93.48	96.74	94.57	93.48	91.30	94.57	90.22	94.46	±2.43
Average IoU (%)	all	**31.22**	28.00	27.89	29.31	27.67	28.08	28.14	27.93	27.96	23.29	27.95	±1.96
	IoU > 0	**32.27**	29.27	28.51	31.36	28.60	29.69	30.11	30.59	29.57	25.81	29.58	±1.77
Average Score (%)	all	**57.65**	52.20	53.07	52.56	55.44	54.18	50.60	54.76	51.24	44.95	52.66	±3.42
	IoU > 0	59.16	54.57	54.04	56.06	57.26	57.25	54.06	**59.61**	54.05	49.34	55.54	±3.01
Average Max IoU (%)	all	**69.02**	63.69	66.47	65.42	64.44	66.35	65.87	62.82	66.51	61.85	65.25	±2.09
	IoU > 0	**71.34**	66.59	67.95	69.99	66.61	70.17	70.47	68.80	70.34	68.56	69.08	±1.65
Average Score @ Max IoU (%)	all	**87.15**	84.07	86.18	85.58	86.39	85.23	83.71	79.99	82.33	81.49	84.21	±2.34
	IoU > 0	90.08	87.89	88.09	**91.56**	89.30	90.12	89.55	87.61	87.06	90.32	89.16	±1.44

**Table 10:** Comparison with other authors models.

Article Reference	Test Method	Model Architecture	Unit	Train/Test Set	Metric Type	Metric Value
[34]	One pass	Mask R-CNN	Patient/Case	241/98	Accuracy	100%
[35]	One pass	Faster R-CNN	Image	5252/620	Sensitivity	87%
Proposed method	10 fold cross validation (avg)	Faster R-CNN	Patient/Case	830/92	Accuracy	94.46%
	10 fold cross validation (best)	Faster R-CNN	Patient/Case	830/92	Accuracy	97.83%

## Data Availability

In this study we used the DukeBC dataset. DukeBC is a public domain dataset owned by Duke Hospital, Durham, North Carolina, USA. This dataset comprises 922 breast cancer patients’ cases MRI-based and can be downloaded at “The Cancer Imaging Archive” website: https://wiki.cancerimagingarchive.net/pages/viewpage.action?pageId=70226903 (accessed on 15 August 2023).
